# Hormone therapy after the Women's Health Initiative: a qualitative study

**DOI:** 10.1186/1471-2296-7-61

**Published:** 2006-10-23

**Authors:** Linda M French, Mindy A Smith, Jodi S Holtrop, Margaret Holmes-Rovner

**Affiliations:** 1 Department of Family Medicine, Michigan State University, East Lansing, Michigan, USA; 2 Department of Medicine, Michigan State University, East Lansing, Michigan, USA

## Abstract

**Background:**

Publication of results from the Women's Health Initiative study in July 2002 was a landmark event in biomedical science related to postmenopausal women. The purpose of this study was to describe the impact of new hormone therapy recommendations on patients' attitudes and decision-making in a primary care practice.

**Methods:**

A questionnaire including structured and open-ended questions was administered in a family practice office waiting room from August through October 2003. Rationale for taking or not taking hormone therapy was specifically sought. Women 50–70 years old attending for office visits were invited to participate. Data were analyzed qualitatively and with descriptive statistics. Chart review provided medication use rates for the entire practice cohort of which the sample was a subset.

**Results:**

Respondents (n = 127) were predominantly white and well educated, and were taking hormone therapy at a higher rate (38%) than the overall rate (26%) for women of the same age range in this practice. Belief patterns about hormone therapy were, in order of frequency, 'use is risky', 'vindication or prior beliefs', 'benefit to me outweighs risk', and 'unaware of new recommendations'. Twenty-eight out of 78 women continued hormones use after July 2002. Of 50 women who initially stopped hormone therapy after July 2002, 12 resumed use. Women who had stopped hormone therapy were a highly symptomatic group. Responses with emotional overtones such as worry, confusion, anger, and grief were common.

**Conclusion:**

Strategies for decision support about hormone therapy should explicitly take into account women's preferences about symptom relief and the trade-offs among relevant risks. Some women may need emotional support during transitions in hormone therapy use.

## Background

A highly publicized reversal in recommendations for menopausal hormone therapy (HT) occurred in July 2002 when results were reported from the estrogen plus progestogen therapy (EPT) vs. placebo arm of the Women's Health Initiative (WHI)[1]. HT is no longer recommended for disease prevention, while use for treatment of menopausal symptoms for limited duration is still acceptable. This was an abrupt reversal in recommendations that represents a unique opportunity to study patient reactions to the change.

A telephone survey conducted among staff model health maintenance organization members in early 2003 [2] identified that women had poor knowledge of the WHI results despite directed mailings about the study findings in July through August 2002, and that a little over half of users' had attempted to stop HT after WHI results were released[3]. One quarter of such women resumed HT use[4,5].

The purpose of this study was to explore how women seeking care in a primary care setting have experienced the impact of the new recommendations on their attitudes and decisions about HT from a qualitative perspective.

## Methods

### Subjects and setting

Since current recommendations discourage use of HT except for symptomatic relief of menopausal symptoms, care-seeking women were the population of interest rather than a community-based sample. The study was conducted in the waiting room of a faculty family medicine clinic serving a predominantly white non-Hispanic population (about 65%) in a small urban/suburban university community. Women aged 50–70 attending for office visits were invited to participate. A priori judgment was that 100 respondents would ensure a broad sampling of the qualitative data. Enrollment was assessed at the end of each month of data collection until that sample size was reached.

### Instrument

A questionnaire including open-ended questions regarding women's HT decisions was developed for this study (See Additional file 1). Questions included current views about HT and change in views after July 2002. Reading level was approximately eighth grade. Pilot testing consisted of obtaining feedback on understandability and acceptability from three women in the appropriate age group with high school education.

### Data collection and analysis

Self-administered questionnaires were located at the reception desk next to the sign-in sheet for office visits. A sign posted above the surveys invited women in the appropriate age range to complete one and return it to the receptionist. A brief explanation of the purpose of the questionnaire was, " to know more about the use of hormone replacement therapy by women in this clinic, and how and why it may be changing". The survey was anonymous and was granted exempt status by the University Committee on Research in Human Subjects of Michigan State University.

Data were analyzed at the conclusion of the data collection period. Numeric codes for descriptive data were assigned and entered into an Access database. Transcribed responses to each free-text question were first analyzed by LMF who generated a list of labels and numeric codes to summarize each concept. A second researcher reviewed the coding scheme for each question for accuracy and completeness. Two independent researchers coded each question using multiple codes when indicated. Disagreements were resolved by discussion. Major themes in the responses were identified by consensus.

A chart review of the electronic medical record for this practice was performed as a measure of comparison between the study sample and the entire clinic population of which the sample is a subset. HT prescribing rates were determined for the whole clinic population of women in the same age range during the study window and during the previous 2 years, based on the presence or not of an estrogen-containing product in the list of active medications.

## Results

### Sample characteristics

There were 133 surveys collected from August through October 2003. Six questionnaires were excluded because year of birth was either missing or outside of the target age range, leaving 127 for analysis. This represents 15% of 847 active female patients born 1933–1952 seen in this practice, and 30% of 419 women of appropriate age with an office visit during the study timeframe.

Demographic characteristics of respondents and health-related quantitative variables are displayed in Table 1. Respondents were mostly non-Hispanic Caucasians and well educated. Thirty-sex percent of survey respondents were currently taking HT. Ninety-four women (74%) had used HT for more than 5 years including 54 (43%) for more than 10 years. The rate of HT use in the entire practice decreased from 49% during the year prior to July 2002 to 26% at the time of the survey.

**Table 1:** Demographic Characteristics of Study Sample (n = 127)

Characteristic	Number	Percentage
Year of birth		
1933–1937	16	12.6
1938–1942	22	17.3
1943–1947	49	38.6
1948–1952	40	31.5
Race/ethnicity*		
White not Hispanic	115	90.6
African American	3	2.4
Hispanic	3	2.4
Asian	1	0.8
Native American	3	2.4
Pacific Islander	2	1.6
Other	3	2.4
Prefer not to say	2	1.6
Education level		
Less than high school	0	0.0
High school graduate	18	14.2
Some college	20	15.7
Bachelor's degree	22	17.3
Postgraduate education	67	52.8
Self-rated health status		
Excellent	39	30.7
Good	64	50.4
Fair	16	12.6
Poor	7	5.5
Missing data	1	0.8
Current hot flashes		
Yes	99	78.0
No	26	20.5
Missing data	2	1.6

* Instructions were to check all that apply

### Belief patterns

Four general patterns of belief were identified from the data analysis, labeled 'use is risky' (n = 86), 'vindication of prior beliefs' (n = 14), 'benefit to me outweighs risk' (n = 10), and 'unaware of new recommendations' (n = 9). Seven questionnaires had little or no content in the open-ended questions.

The predominant pattern of belief was that HT 'use is risky'. Samples of statements in this category were, "They're not as safe as I thought." "They are not good for you. They increase chances for cancer." "Hormones seem to add to possible cancer problems." "They do not protect the heart, more studies are needed, increased risk in usage." Expressions of fear/concern about personal use of HT were present in ten questionnaires. Sample statements are, "Worried about long term health effects." "I am afraid to take ERT."

The next most common pattern was 'vindication of prior beliefs'. Sample statements were "Though I took them for awhile I was never totally comfortable with the idea." "Recent studies have confirmed my feelings that HRT is not without its own set of risks." "If menopause is a normal process, why do I need to take drugs for it?" "I am a firm believer in going as natural as possible and everything I have read indicated I am on the right track."

In the category of 'benefit outweighs risk' examples are "I believe the risk is less than the benefits." "After reading and discussing the reports on their adverse effects, I don't think I'm at risk for any of the adverse health effects". " I know I am taking a risk by taking them (breast cancer). [I take them] so that I feel like myself and not the wicked witch."

A few women did not give evidence that they knew of any recent change in recommendations or new research findings. Examples are, "Hormone therapy is necessary for women for its effects emotional and physical." " [I] Believe they are helpful in maintaining hormone levels during perimenopause." "They make me feel good and I have no ill effects."

Some women were misinformed, believing that HT provides no health benefits even for bone strength, while the WHI findings did confirm decreased risk for fractures and colon cancer with HT.

Although we did not ask if women felt confused, six spontaneously expressed confusion or uncertainty such as "I am confused" "I don't know what to do." " I don't know what to believe."

A few women seemed to express anger. Sample statements were, "I wish I had never taken HRT!" "...and we were lied to by the manufacturers?!"

### Subgroup analysis based on hormone use

Analysis was performed based on category of experience with HT use into never-users, former users (quit before July 2002), quitters (quit after July 2002 and not currently using), restarters (quit after July 2002 but restarted and currently using), and continuers (did not quit after July 2002 and currently using). Six questionnaires had missing or contradictory responses precluding determination of whether they were former users or quitters. Characteristics of these subgroups are displayed in Table 2.

**Table 2:** Characteristics of Respondents by Category of HT Use Experience

Category of HT use	Portion of sample entire sample*		More than 5 years use		More than 10 years use		Estrogen only use		Good or excellent self-rated health		Moderate or severe hot flashes now		Moderate or severe hot flashes at worst ever	
	N	%	N	%	N	%	N	%	N	%	N	%	N	%
Never users	18	14	0	0	0	0	0	0	15	83	6	33	13	72
Former users^+^	25	20	10	40	2	8	9	43	15	64	5	20	17	68
Quitters^#^	38	30	26	68	10	26	8	21	32	84	15	39	27	71
Restarters^&^	12	9	9	75	6	50	4	33	7	58	6	50	11	92
Continuers^	28	22	21	75	12	43	7	25	26	93	1	4	20	73

* Total less than 100% due to 6 responses with missing data to determine whether they were former users or recent quitters.

^+ ^Stopped hormone therapy prior to July 2002.

^# ^Stopped hormone therapy after July 2002, and not using at the time of response.

^&^Stopped hormone therapy after July 2002, and resumed use, using at time of response.

^ Respondents using HT at the time of response and that did not stop hormone therapy after July 2002.

There were 43 (34%) respondents who were not using HT in July 2002. Never-users (n = 18) were largely represented by the vindicated belief pattern. Some described personal medical reasons for not using. Former users (n = 25) sometimes gave reasons for stopping based on side effects of HT. Also in this group were women who used HT for a while based on physician recommendation, though never comfortable with the idea. Others stopped because they had no perceived need to continue.

Recent quitters (n = 38) were a highly symptomatic and concerned group. "My hot flashes have returned with nearly the same intensity they were when I first started using hormones. I am consulting with my doctor now to find out what my options are." "I miss the effects of estrogen – positive attitude, more energy, higher metabolism and sex drive." "I am not taking hormones now solely because I fear the side effects, I felt much better when I was taking them." Some women were considering restart.

Restarters (n = 12) were also symptomatic and typically felt unable to quit, at least not yet. The ages of these women were relatively even across the span. Of these respondents five had changed hormone therapy to a lower dose or different formulation. Sample quotes are "Have reduced the number of days on/off to see if I can stop altogether. (not yet)" "Am tapering off, however hot flashes and mood swings make it difficult."

Continuers (n = 28) were a relatively young and healthy subgroup, yet the majority had used HT for more than 5 years. Most (59%) also answered yes to the question, 'Has your opinion about hormone therapy changed?' and 39% had changed dose or formulation. Predominantly, women expressed a need for symptom relief as rationale for their choice. "I take hormones because I feel better – I can sleep and work without hot flashes." "I attempted gradual reduction and had significant increase in hot flashes." Nevertheless, fears about long-term use were present in this group of women. "I am now concerned about the long-range effect after 17 years of taking them." "Worried about long-term health effects. Wasn't aware of any when I started therapy 15 years ago."

## Discussion

These findings show that women have largely accepted the idea based on recent research findings that HT use is risky. It might be expected that controversy about HT decisions is abating as women stop using HT and/or never start. However, the importance of symptom relief to peri- and postmenopausal women should not be underestimated.

Surveys of nationally representative samples of menopausal women conducted in the 1990's found that 54% had taken hormone therapy (HT) at some time in the perimenopausal years, with 32% for at least 5 years[6], and that 38% of women aged 50–70 were currently taking HT[7]. Rates as high as 50% and 85% of women taking HT for at least 5 years and 2 years respectively, were reported in one affluent, predominantly white, suburban community[8]. The hormone use of women receiving care in this practice prior to report of WHI results seems to approximate the latter, community-based sample.

Prior qualitative studies on menopause and HT have provided useful information about women's views. Women may believe that sometimes menopause requires medical attention, but is primarily a marker or symbol of general life-stage[9]. Although aging can be troubling to women, even initially pessimistic women often reappraise the menopause transition as a time of personal growth[10]. One New Zealand study[11] found that women utilize a full range of socially available interpretations of HT as a medicine, beauty product, drug (of dependence), and poison. The decision to begin HT is affected by several spheres of influence including internal, interpersonal relationships, external (i.e. cultural, such as ageism and sexism), and perceived consequences of the treatment decision[12]. A study[13] of HT decision-making has also demonstrated that presentations about risk are interpreted within an individual's belief system. This study adds insight into the dimension of reversal in medical recommendations.

Some quitters seemed to express grief. Others who are still using appear to feel guilty about it, as if it were a vice. We were impressed by the degree of emotion we perceived in the written responses of these women. It was evident in their choice of words as well as emphases given through the use of large font, underlining, and exclamation points.

Our findings are consistent with the survey by Grady et al, showing that the major predictor of resuming HT was troublesome withdrawal symptoms[5]. The expressions of emotion including worry and anger in this qualitative data among women who quit, or tried to quit, taking HT after July 2002 were not captured in prior studies. It is not clear to women that the recommendation not to use HT for disease prevention and health promotion is different from a recommendation not to use at all, even in the face of troublesome symptoms.

Few studies have been undertaken to assess women's quality of life with menopausal symptoms. Those few have found an impact similar to that found in other studies of advanced heart failure[14,15]. While no overall benefit for quality of life was found in the WHI[16], participants were a relatively asymptomatic population. On the other hand, women seen in primary care practice on HT seem to be a highly symptomatic group of women. A decision analysis by Kim et al in 2003 concluded that with even a tiny improvement in quality of life with EPT, the benefits of therapy for five years would outweigh the risks for most women[17]. The difficulty of decision-making for individual women is more complicated. Support for difficult medical decisions should include individualized risk communication[18]. Minelli et al performed a decision analysis concluding that modeling should be tailored to individual women to be useful in clinical practice[19]. Optimal decision-making about use of HT has long been known to be influenced by women's preferences for health states, and is thus 'preference-sensitive'[20]. Women's preferences about the trade-off between long term risk and present symptom relief is the most relevant aspect now and its assessment is complex. Tools for decision support in the revised context of scientific evidence are lacking.

It might be expected that women in our sample choosing to use HT after results from the WHI report about EPT would be mainly women taking estrogen only. However, among both restarters and continuers the women taking estrogen only were a minority. The results of the estrogen-only therapy vs. placebo portion of the WHI was reported in April 2004 and showed a non- significant excess of 2 events per 10,000 women years[21]. The present study was conducted prior to availability of those results, so their potential impact on patients' attitudes could not be assessed. Those results did not receive the kind of publicity as the 2001 report, and women are therefore less likely to know about them.

### Study limitations

The study sample was not entirely representative of this practice, since women who used HT were over sampled. In addition, the majority of the sample was white and highly educated. It is likely that these same women are more distressed by change in HT recommendations. While a sample size of 127 is small for a quantitative survey it is large for a qualitative study.

Identifying women based on the prescription of an estrogen containing medication may inflate the numerator for the practice-based rate of HT use, since not all women prescribed HT are likely to be taking it. Nevertheless, concerns of participants in this study may well represent those of the large number of women who have either quit HT or continue to use it. Future studies should systematically recruit more women from race/ethnic groups other than white non-Hispanics to clearly capture their perspectives.

Written answers to open-ended questions for qualitative data collection is not as rich as interview or focus group techniques. Results obtained suggest the need for further qualitative research using those methods, as well as quantitative surveys of primary care and general population women.

## Conclusion

This sample of care-seeking women remains concerned about pros and cons of HT. Strategies for decision support regarding HT should explicitly take into account women's preferences about symptom relief and trade-offs among relevant risks. The discussion about such trade-offs is complex and would benefit from the availability of new decision support tools. They need accurate information about how research results apply to them. Our findings suggesting strong emotional overtones to decision-making about HT leads us to believe that many women are also likely to need emotional support in their transition off or restart of hormones in order to limit their experience of suffering and/or guilt.

## Abbreviations

HT – hormone therapy

EPT – estrogen and progestin therapy

ET – estrogen-only hormone therapy

## Competing interests

None of the authors has financial or non-financial competing interests regarding this study.

Each author contributed to the study design and analysis including the design of the instrument used. Each also contributed to the written report.

## Pre-publication history

The pre-publication history for this paper can be accessed here:



## Supplementary Material

Additional file 1 Survey Instrument Click here for file
