# Supplementary material for: Hormone therapy after the Women's Health Initiative: a qualitative study
Source: BMC Fam Pract. 2006 Oct 23;7:61. doi: 10.1186/1471-2296-7-61 (PMC1634847; doi:10.1186/1471-2296-7-61)
Supplement: Additional file 1 — Survey Instrument [file 1471-2296-7-61-S1.doc]

# Survey

# Hormone Replacement Therapy Survey

We would like to know more about the use of hormone replacement therapy by women in this clinic, and how and why it may be changing. No information is requested that identifies individual women. It is anonymous. The survey will take about 3 minutes. Please return it to the receptionist before you leave. The results of the survey will be available here at this office in a few months. You indicate your voluntary agreement to participate by completing and returning this questionnaire.

If you already have completed this survey another day, please return the survey and check here. ❑

If you do not wish to participate, please return the survey. Please let us know your reason on the line below. ­­­­

­­­_________________________________________________________________­­

1. Have you ever taken estrogen and/or progestin medicines for menopause? ❑ No ❑ Yes

If yes:

- 1. Why did you start?
  2. What is the total amount of time you have taken hormones for menopause even if you stopped for a while?

❑ Less than 1 year

❑ 1-4 years

❑ 5-9 years

❑ 10 years or more

1. What type of hormones have you taken for menopause? (check all that apply)

❑ Estrogen only

❑ Progesterone only

❑ Both estrogen and progesterone

❑ Other ________________

❑ Don’t know

c. Are you taking hormones for menopause right now? ❑ Yes ❑ No

d. Did you stop taking hormones in the last year? ❑ Yes ❑ No

If yes, why?______________________________________________________

e. Have you changed your hormones in the last year ❑ No ❑ Yes

If yes, how?______________________________________________________

f. Did you restart hormones in the last year after stopping?

❑ No ❑Yes

2. Where do you learn about hormone therapy? (check all that apply)

❑ Television ❑ Family

❑ Magazines or newspapers ❑ Friends

❑ Radio ❑ Doctors

❑ Internet ❑ Nurses or physician assistants

❑ Other ____________________________

PLEASE GO TO NEXT PAGE

3. Has your opinion about hormones changed in the last year? ❑ Yes ❑ No

If yes, how?______________________________________________________________

4. Please answer the question appropriate for you.

If you **are** taking hormones right now, what are the reasons you take the hormones?

If you are **not** taking hormones right now, what are the reasons you do not take them?

5. What have you heard on TV or radio or read in magazines about hormones in the last year?

6. How would you rate your health right now?

❑ Excellent ❑ Good ❑ Fair ❑ Poor

7. Do you have hot flashes now?

If yes, how bothersome are they to you?

❑ Not at all ❑ Mild ❑ Moderate ❑ Severe

8. At their worst, how bothersome have your hot flashes ever been?

❑ Not at all ❑ Mild ❑ Moderate ❑ Severe

9. What year were you born? (please fill in the date) Year: 19___ ___

10. What is your race/ethnicity? (check all that apply)

❑ African American ❑ Native American

❑ Asian ❑ Pacific Islander

❑ Hispanic ❑ White, not Hispanic

❑ Other, none of the above ❑ Prefer not to answer

11. What is the highest level of education you have completed? (check one only)

❑ Less than high school ❑ Bachelor’s degree

❑ High school ❑ Postgraduate degree

❑ Associate’s degree or vocational training

Please use the space below for any comments you have. Thank you for your time in filling out this survey!
